# EHMTI-0022. Pharmacokinetic variability of drugs used for prophylactic treatment in migraine

**DOI:** 10.1186/1129-2377-15-S1-G36

**Published:** 2014-09-18

**Authors:** P Pavbro, P Tfelt-Hansen

**Affiliations:** 1 University of Copenhagen, Danish Headache Center in Glostrup, Glostrup, Denmark

## Introduction

In order to evaluate pharmacokinetic variability of preventive migraine drugs we have in this study reviewed single dose studies.

## Methods

PubMed was searched for each drug with the “drugs generic name”, “pharmacokinetics”, and “single dose”. Variability was calculated as coefficient of variation (CV).

## Results

A total of 105 single-dose kinetic studies were reviewed but only a few representative results are presented:

Extended release propranolol 160 mg ( n = 2): CV for Cmax = 45-55%, and CV for AUC: 43-48%. Propranolol 80 mg (n =1): Ratio for Cmax = 14 and ratio for AUC = 24. Metoprolol 100 mg (n = 2): CV for Cmax = 23-64%, and CV for AUC: 26-75%. Metoprol 100 mg (n = 1): Ratio for Cmax = 8 and ratio for AUC = 23. Extended release divalproex 500 and 1000 mg ( n = 2): CV for Cmax = 12-21%, and CV for AUC: 19-30%. Topiramate 100 mg ( n = 1): CV for Cmax = 16% and CV for AUC = 14%.. Candesartan 16 mg ( n =2) : CV for Cmax = 31-34% and CV for AUC 26-28%.

## Conclusion

A coefficient of variation of a pharmacokinetic parameter above 40% is considered to be high . The results for the AUCs of propranolol and metoprolol show high variability; and it is not likely that the migraine patients can be treated with a fixed dose-schedule with these two drugs. Instead dosing should in each case be tailored to the individual migraine patients.

No conflict of interest.

